# Endodontic Management of Middle Mesial Canal in Mandibular Molars: A Report of Three Cases

**DOI:** 10.7759/cureus.35826

**Published:** 2023-03-06

**Authors:** Pradeep Solete, Delphine P Antony, Lokhasudhan Govindaraju, Sowmya Kavoor, Hima Sandeep A, Majety Sharath Chandragupta

**Affiliations:** 1 Conservative Dentistry and Endodontics, Saveetha Dental College, Chennai, IND; 2 Conservative Dentistry and Endodontics, Sri Ramachandra Institute of Higher Education and Research, Chennai, IND; 3 Laser Dentistry, Dr.Gupta’s Dental Specialities Center, Chennai, IND

**Keywords:** root canal obturation, root canal therapy, middle medial canal, endodontics, mandibular molar

## Abstract

Knowledge of both normal and aberrant root canal anatomy is the key to any successful root canal treatment. A mandibular molar demonstrates considerable variations with respect to additional canals or roots. The clinician must aim to identify all possible canals with the help of any magnification aids. This report discusses the variations of the middle mesial canal in mandibular molars.

## Introduction

The prerequisite of any endodontic therapy is to have familiarity with the root canal anatomy of the tooth. The pulpal cavity is the most complex structure in nature. Identifying all the portals of exit, debridement of the entire root canal system, and sealing all the exits with three-dimensional filling materials make endodontic treatment successful [1].

Clinically, a mandibular molar has many anatomical variations, like the presence of additional roots in the distolingual or mesiobuccal aspect, C-shaped canal anatomy, and three canals in mesial or distal roots including a middle mesial canal (MMC) or middle distal canal (MDC), etc [2-5].

The incidence of having an extra canal in the mesial root is 1-15% [6]. The MMC can be described as a bleeding spot existing within the grooves, between the mesiobuccal (MB) canal and mesiolingual (ML) canal. The orifice size is smaller when compared to the other canals [3]. Pomeranz et al., in 1981, classified the MMC into three types, namely: A fin, A confluent, and an independent type [2]. The MMC can also merge with the MB or ML canals, at the middle third of the root canal system.

For the identification of this MMC, one must rely on multiple radiographs, cone beam computed tomography (CBCT), and another form of magnification aid such as magnifying loupes or dental operating microscopes in order to limit procedural errors during exploration of the third canal [7].

 This paper reports three cases with different variations of MMC in the mandibular first molar.

## Case presentation

Case 1

A 23-year-old male patient presented with a complaint of pain in a left lower back tooth for the past one week. The pain increased on taking cold foods and at night. Clinical examination revealed the presence of deep caries with pain on probing. On radiographic examination, the presence of two roots and radiolucency involving the distal pulp horn of the left mandibular first molar no 36 were seen. The clinical and radiographic findings led to the diagnosis of dental caries with symptomatic irreversible pulpitis in 36.

Protocol for Treatment

Informed consent was obtained from the patient before undergoing treatment. Inferior alveolar nerve block (IANB) was administered using 2% lignocaine hydrogen chloride (HCl) with 1:2 lacs units of adrenaline (Lignox, Indoco Remedies Ltd, Mumbai, Maharastra, India). The access cavity was prepared by endodontic access bur size 2 (Dentsply-Sirona, Charlotte, North Carolina, United States) under rubber dam isolation. The access cavity was refined using Endo-Z bur (Dentsply-Sirona), exploration of the pulp chamber was done using an endodontic explorer, and the canals were negotiated using a size 10 K file (Mani, Inc; Tochigi, Japan). Four canals were initially located, two mesial and two distal.

In order to enhance the magnification, an OPMI® pico dental operating microscope (Carl Zeiss AG, Oberkochen, Germany) was used. The bleeding spot was identified between the MB and ML orifices. Ultrasonic tips (Start-X, Dentsply-Sirona) were used to explore the MMC orifice and the canal was negotiated using a size 10 K file. The working length was determined by a ProPex Pixi Apex locator (Dentsply-Sirona). Finally, a total of five distinct orifices were identified: Mesially three (MB, middle mesial, ML) and distally two (distobuccal, distolingual).

**Figure 1:**
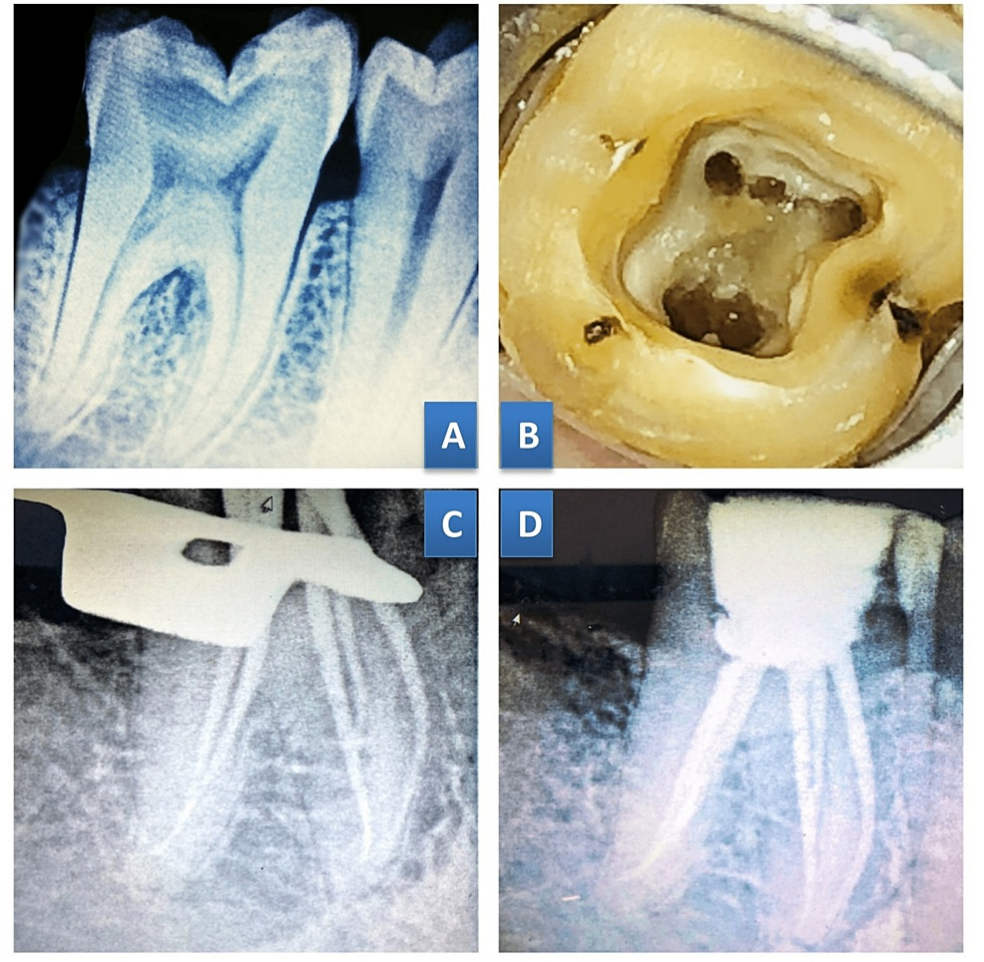
Case 1. Middle mesial canal merged with mesiobuccal canal at the middle third region in 36 (A) Preoperative radiograph;  (B) Access cavity preparation; (C) Master cone radiograph; (D) Post-obturation radiograph

The shaping and cleaning were carried out by ProTaper Universal NiTi rotary instrument (Dentsply-Sirona) in a crown-down manner as all the canals were wide including the MMC. Irrigation was carried out using 3% sodium hypochlorite solution (Prime Dental Products Private Limited, Thane, Maharastra, India) and Endoprep-RC (Anabond Stedman Pharma Research Pvt Ltd, Chennai, Tamil Nadu, India). Canals were dried using paper points and obturation was done using warm vertical compaction with resin sealer (AH Plus sealer, Dentsply-Sirona). Post-obturation radiograph revealed that MMC merged with the MB canal at the middle third region. The access cavity was restored using a composite restoration (3M™ ESPE Filtek™ Bulk Fill, 3M, St. Paul, Minnesota, United States) (Figure 1).

 Case 2

**Figure 2:**
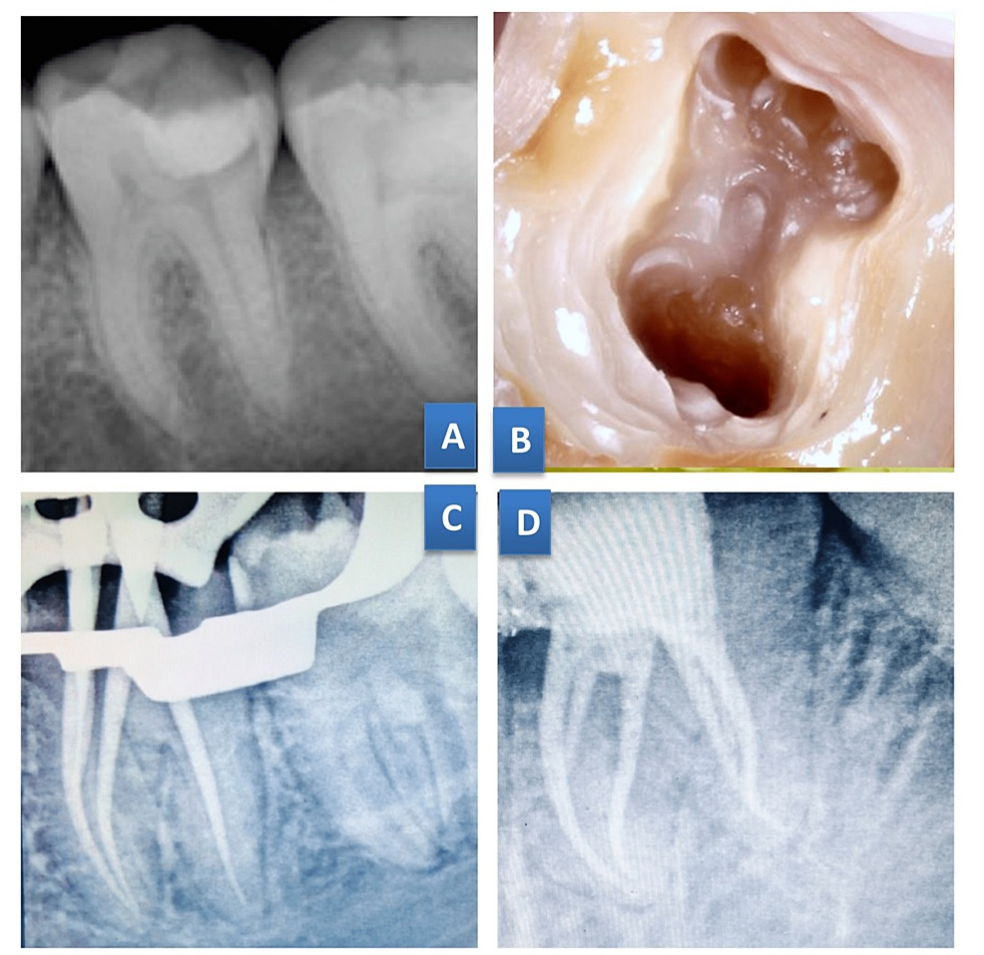
Case 2. Middle mesial canal merged with mesiolingual canal at the middle third region in 46 (A) Preoperative radiograph;  (B) Access cavity preparation; (C) Master cone radiograph; (D) Post-obturation radiograph

A 28-year-old male patient presented with a complaint of sensitivity and discomfort on a right lower back tooth for the past two weeks. Caries excavation was done followed by a pulp capping procedure using dycal and intermediate restorative material in the right mandibular first molar no. 46. The pain increased on the third postoperative day of the procedure. Radiographic examination revealed the presence of radio-opaque filling material involving the pulp in 46. The clinical and radiographic findings led to the diagnosis of failed pulp capping with symptomatic irreversible pulpitis in 46. The treatment protocol followed, was the same as explained in Case 1. In this case, five distinct orifices were identified: three mesially (MB, MMC, ML) and distally two (distobuccal, distolingual). Post-obturation radiograph revealed that MMC was merged with the ML canal at the middle third region. The access cavity was restored using composite restoration (3M ESPE Filtek Bulk Fill) (Figure 2).

Case 3

**Figure 3:**
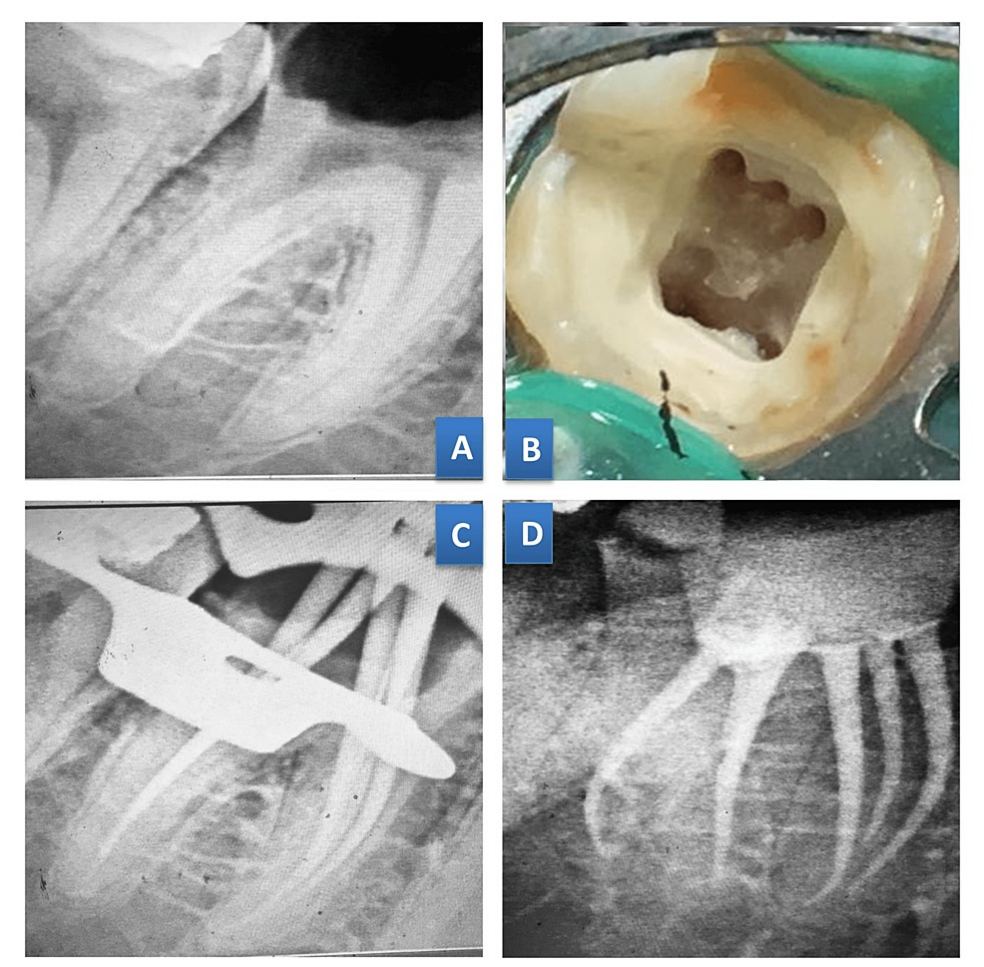
Case 3. Independent middle mesial canal with radix entomolaris 46. (A) Preoperative radiograph;  (B) Access cavity preparation; (C) Master cone radiograph; (D) Post-obturation radiograph

A 34-year-old male patient presented with a complaint of decay and dull pain in a right lower back tooth for the past two months. Clinical examination revealed loss of tooth structure with pain on percussion and periapical palpation in tooth number 46. Radiographic examination revealed the presence of additional roots between mesial and distal roots, with loss of enamel and dentin. The clinical and radiographic findings led to the diagnosis of dental caries with asymptomatic irreversible pulpitis and symptomatic apical periodontitis in 46. The treatment plan was a multi-visit root canal treatment. The protocol for the treatment was the same as that followed in Case 1. In this case, too, five distinct orifices were identified: mesially three (MB, MMC, ML), distally one, and distolingually one orifice suggestive of radix entomolaris. After completion of cleaning and shaping, an intracanal medicament (calcium hydroxide) was placed and the cavity was sealed with intermediate restorative material (IRM). During the second visit, the final shaping was done using ProTaper F2 and obturation was done using warm vertical compaction with resin sealer (AH Plus sealer). Post-obturation radiograph revealed independent MMC and radix entomolaris in 46 (Figure 3).

## Discussion

Many authors have reported the existence of additional roots or canals in mandibular molars [8,9]. Critical evaluations of multiple angulated radiographs help the operator better plan the endodontic treatment. A study done by Chavda et al. showed that magnification predominantly helped in identifying the thin MMC, along with ultrasonics [10]. MMC are very tiny and may be present deep into the isthmus; however, the use of a dental operating microscope at higher magnification makes it easier in locating them [11]. Ultrasonic tips will be useful in troughing the area between MB and ML orifices and careful negotiation has to be done using smaller files [12]. The ultrasonic tip eliminates the use of a conventional handpiece, as the size of the head will obstruct the field of vision. Any instrumentation close to the furcation area should be used judiciously. The geometry of this MMC is hourglass-shaped; the preparation at the midsection will be closer to the danger zone [11,12].

Based on the Pomeranz et al.'s classification, an independent canal implies that it originated separately between MB and ML orifices and terminated as a separate foramen, which is found to be rare [2]. In our report, Case 3 had an independent type whereas cases 1 and 2 had a confluent type.

The uniqueness of Case 3 is the presence of an MMC with radix entomolaris. This tooth had three roots and five canals, i.e., mesial root (three canals: MB, MMC, ML), distal two roots (distal and radix entomolaris) had one canal each. The existence of additional roots is unusual but does occur. The additional root, which was found distolingually, is called "radix entomolaris”, a name given by Carabelli et al. [13]. The radix can further be classified based on the root/canal curvature, proposed by Ribeiro et al. [14]: Type 1, straight root/root canal; Type 2, initially curved entrance followed by straight root/root canals; and Type 3, curvature at coronal third followed by buccally oriented curvature starting from middle to apical third. In Case 3 of this report, Type 2 radix entomolaris was seen.

In Case 1 and Case 2, the mesial roots had canals with type XV Sert and Bayirli classification [15] (3-2) where the MMC merged with the middle third of either MB or ML canals, respectively. In Case 3, the mesial root had a separate MMC with type VIII of Vertucci classification [16] (3-3), and all three canals ended with separate foramen.

 In a study conducted by Sherwani et al. in 2016 in the Indian population, 67% of MM orifice was found between MB and ML, 20 % was closer to ML orifice and 12% was closer to MB orifice [17]. Nosrat et al. stated that the MMC was closer to the ML orifice most of the time, followed by the middle region on MB and ML orifices [18]. Toubes et al. stated that the MMC orifice was closer to MB (46%), followed by ML (31%), and found separately between MB and ML (23%) [19].

The highly variable morphology of mandibular molars indicates that careful examination is mandatory. Failure to recognize the additional canals leads to unsuccessful treatment with persisting symptoms [20]. The use of the dental operating microscope, ultrasonics, and CBCT is invaluable in treating all these aberrant morphologies undergoing endodontic therapy.

## Conclusions

An understanding of the complex anatomy of the tooth is the key to the success of endodontic treatment. The varied location and configuration of the MMC make canal preparation and obturation more challenging. The careful interpretation of the radiograph, use of magnification, latest instruments, proper irrigation protocol, and sealing of all portals of exit leads to successful treatment.
